# Bacterial Deposition of Gold on Hair: Archeological, Forensic and Toxicological Implications

**DOI:** 10.1371/journal.pone.0009335

**Published:** 2010-02-19

**Authors:** Genevieve Phillips, Frank Reith, Clifford Qualls, Abdul-Mehdi Ali, Mike Spilde, Otto Appenzeller

**Affiliations:** 1 Fluorescence Microscopy Facility, Cancer Research and Treatment Center, University of New Mexico, Albuquerque, New Mexico, United States of America; 2 Centre for Tectonics, Resources and Exploration, School of Earth and Environmental Sciences, The University of Adelaide, North Terrace, South Australia, Australia; 3 CSIRO Land and Water, Environmental Biogeochemistry, PMB2, Glen Osmond, South Australia, Australia; 4 Department of Mathematics and Statistics, University of New Mexico, Albuquerque, New Mexico, United States of America; 5 Department of Earth and Planetary Sciences, University of New Mexico, Albuquerque, New Mexico, United States of America; 6 Institute of Meteoritics, University of New Mexico, Albuquerque, New Mexico, United States of America; 7 New Mexico Health Enhancement and Marathon Clinics Research Foundation, Albuquerque, New Mexico, United States of America; University of Hyderabad, India

## Abstract

**Background:**

Trace metal analyses in hair are used in archeological, forensic and toxicological investigations as proxies for metabolic processes. We show metallophilic bacteria mediating the deposition of gold (Au), used as tracer for microbial activity in hair *post mortem* after burial, affecting results of such analyses.

**Methodology/Principal Findings:**

Human hair was incubated for up to six months in auriferous soils, in natural soil columns (Experiment 1), soils amended with mobile Au(III)-complexes (Experiment 2) and the Au-precipitating bacterium *Cupriavidus metallidurans* (Experiment 3), in peptone-meat-extract (PME) medium in a culture of *C. metallidurans* amended with Au(III)-complexes (Experiment 4), and in non-auriferous soil (Experiment 5). Hair samples were analyzed using scanning electron microscopy, confocal microscopy and inductively coupled plasma-mass spectrometry. In Experiments 1–4 the Au content increased with time (*P* = 0.038). The largest increase was observed in Experiment 4 *vs.* Experiment 1 (mean = 1188 vs. 161 µg Kg^−1^, Fisher's least significance 0.001). The sulfur content, a proxy for hair metabolism, remained unchanged. Notably, the ratios of Au-to-S increased with time (linear trend *P* = 0.02) and with added Au and bacteria (linear trend, *P* = 0.005), demonstrating that larger populations of Au-precipitating bacteria and increased availability of Au increased the deposition of Au on the hair.

**Conclusion/Significance:**

Interactions of soil biota with hair *post mortem* may distort results of hair analyses, implying that metal content, microbial activities and the duration of burial must be considered in the interpretation of results of archeological, forensic and toxicological hair analyses, which have hitherto been proxies for *pre-mortem* metabolic processes.

## Introduction

The analyses of heavy- and trace metal and metalloid content of hair, such as arsenic (As), copper (Cu), lead (Pb), zinc (Zn), gold (Au), nickel (Ni), cadmium (Cd) and mercury (Hg), is an established monitoring technique for environmental exposure of human populations [1]. Such analyses have also been used in criminal cases leading to convictions based on excessive levels of toxins.

In 1826, long before the advent of modern analytical methods, evidence for high levels of As was found in tissues of a murdered man. His wife and lodger were convicted of murder and sentenced to death by hanging but only the lodger was executed. The wife was pardoned because of the “concern of the trial judge as to the implications of the toxicological evidence presented at trial” [2]. Modern methods of hair analysis have done little to lessen the numbers of overturned judgments on murder convictions based on excessive levels of As in hair found *post mortem*. For example, a recent story in the *UNION-TRIBUNE* of San Diego (2008) reports on a woman convicted of murdering her husband with As, because of the high levels of the toxin in his hair found after death. However, on appeal, the woman was released after her conviction was found to have been based on toxicological hair analyses and judgmental errors.

Hair analysis was also used to try and resolve the debate about whether or not Napoleon's death was a result of As poisoning [3]. Suggestions that the high As levels found in his hair were due to contamination after his death and not due to the As, he received as treatment while ailing, have been put to rest by the discovery of high concentrations of other metals in his hair. These included Hg and antimony (Sb), both given as part of accepted medical treatments at the time in the form of calomel (salt of Hg), tartar emetic (Sb) in addition to As containing mediations [4].

Hair has also been used to reconstruct diet and life styles of ancient civilizations, such as food sources of indigenous peoples living in the Aleutian Islands [5] and in the reconstruction of ritual child-sacrifices by the Incas centuries ago [6]. High levels of As in hair, found recently, in mummified infants have been linked to the beginnings of mummification practices by the Chinchorros, a South American people, 8000 years ago [7]. The hypothesis proposed to explain the high levels is that As poisoning from contaminated water lead to high miscarriage rates in the mothers, which in turn resulted in “grief-responses” by the parents, culminating in efforts to preserve their dead offspring and thus to the earliest known artificial mummification [7].

A study of a pre-Hispanic mummified head (AD 1418–1491, cast doubt on the assumption of hair analyses as one means to reconstruct *pre-mortem* metabolism and for the detection of toxic elements accumulated in life [8]. This study suggested that microbial activity in South American soils might have led to the deposition of large quantities of toxic metals (As 38 µg g^−1^, average range in humans 0.03–3 µg g^−1^; Pb 8680 µg g^−1^, average range in humans 5–29 µg g^−1^; Hg 6410 µg g^−1^ average range in humans 0.4–1.2 µg g^−1^of hair). These levels are considered incompatible with survival; they, therefore, must have been deposited after death [8].

The levels of toxins (As amongst them) detected *post-mortem* in hair buried in soils have hitherto been assumed to preserve a record of *pre-mortem* metabolism of the subject, and hence to reflect the levels of toxins ingested or otherwise absorbed [9].

Substantial research has ensured that sampling, washing and analyses procedures do not distort the results of the analyses [10]. However, the main issue for hair that has been buried for lengthy periods, even millennia, seems to have been entirely overlooked. This is the possibility of metal deposition on hair, after burial, which is mediated by microbial processes.

Microbes pervade the Earth's crust to depths of several kilometers [11], and are paramount in cycling of many trace and heavy metals and metalloids including Cu, Zn, Cd, Hg, As, Se, Te, U and V [12]. Recent studies of microbial activities in Australian gold mines have shown that microbiota drive a biogeochemical cycle of Au by mediating its solubilization, dispersion, precipitation and biomineralization [13]. A key role in this cycle of Au is occupied by a metallophillic bacterium, *Cupriavidus metallidurans*, which thrives in environments containing high concentrations of mobile heavy metal ions and complexes. *C. metallidurans* is commonly associated with Au grains from placer deposits and contributes to their formation [13].

The suggestion, previously made, that “the plural of anecdote is no data” [14] has led us to conduct the present study. Here we aimed to assess if bacterial activity can lead to the deposition of metals in hair that cannot be removed by the usual washing procedures and provide data that may influence the results of archeological, forensic and toxicological investigations.

We describe experiments to assess if measurable concentrations of Au can be deposited by the bacterium *C. metallidurans* in hair of a modern human in a period of six months after burial in soil. We chose Au in preference to other metals, because it is not usually deposited in significant quantities by metabolic processes in living humans, thus it is a good tracer of microbial activities that add metals *post mortem*. Additionally, while Au cycling in the environment has been shown to occur, its turnover, is lower than for other metals [13] Hence, Au is assumed to be a conservative tracer of bacterial activity.

## Results

For a schematic overview of the experimental setup and sampling times in Australia and USA see, Fig. S1.

**Figure 1:**
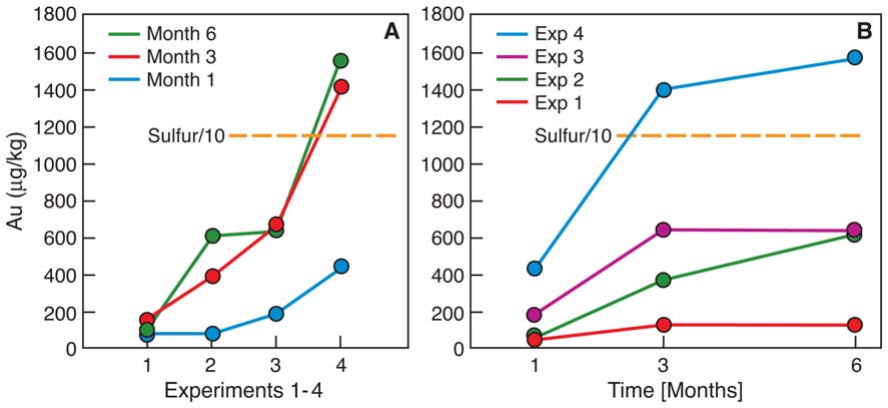
Gold and sulfur content of human hair obtained by ICP-MS analysis. Panel A shows Au and S content **by experiment** (Experiment 1–4). With each experimental increment in Au and bacterial content of the soil (Experiment 1–3) or improved growth conditions (experiment 4 = hair + Au + bacteria in growth medium) the amount of Au increased significantly; main effect (P = 0.009). Panel B illustrates the changes in Au and S contents **with time**. Over the six months duration of burial the Au content in experiment 1 (experiment 1 = hair buried in Australian auriferous soil without the addition of Au or bacterial cells) did not change. However, in all other experiments (Experiments 2–4) there was an increased Au content with time of burial (*P* = 0.038). In *post hoc* testing the Au deposited on the hair in auriferous soil was significantly higher at months 3 and 6 than at month 1 (means = 695, 787, 239 in (µgKg^−1^), respectively) and it was significantly higher in experiment 4 (hair incubated in 500 mL growth medium, inoculated with *C. metallidurans* cells, with added 3 µL of 0.5 M AuCl_4_^−^) compared with experiment 1 (mean = 1188 vs. 161 in (µgKg^−1^), Fisher's least significant difference *P* = 0.001). The S content did not change. Graphic constraints required that the S values be divided by 10.

Overall the Au content in hair increased with time in all experiments conducted with auriferous Australian soils (*P* = 0.038) and in the peptone-meat-extract (PME) medium incubations (main effect (*P* = 0.009); Experiments 1 to 4; Fig 1.). We observed an increasing trend in Au content in hair buried in Australian gold mine soil without further amendment for up to six months (Experiment 1, Fig. 1). However, values were not statistically significant (Fig. 1; table S1.). In Experiments 2 and 3 concentrations of Au deposited in the hair in auriferous soil was significantly higher at months three and six than after one month (means = 695, 787, 239 µg Kg^−1^, respectively), and even higher in Experiment 4 (compared with Experiment 1 (mean = 1188 vs. 161 µg Kg^−1^; Fisher's least significant difference *P* = 0.001) Fig. 1. In the control experiments (Experiment 5) in which hair was buried in non-auriferous soil in the USA, the minute concentrations of Au, present in the hair before burial, remained unaltered after three months of burial (table S1). In Experiments 3 and 4 the highest rate of Au deposition was observed between months zero and three, with a much smaller rate of deposition between months three and six.

**Figure 2:**
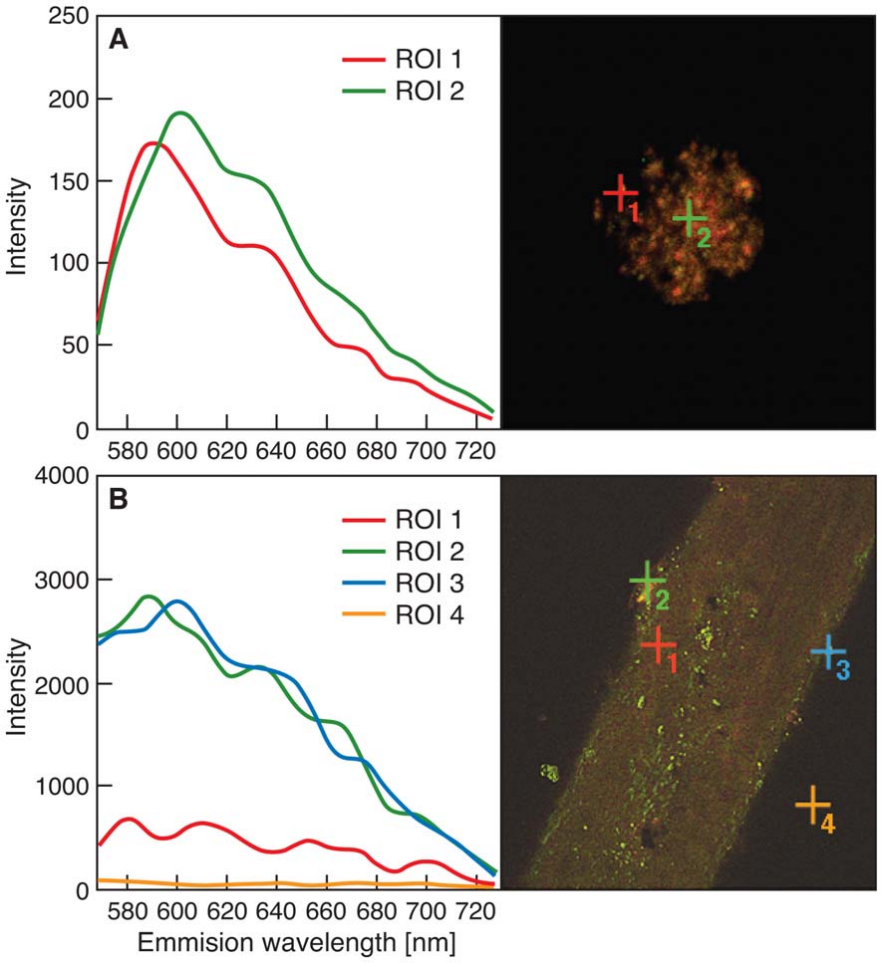
Colloidal gold and gold on a hair shown by confocal microscopy using spectral information of pixel location. **A.** Spectral image of a cluster of 10 µm colloidal Au, displayed as “Lambda Coded”. The spectral information of pixel location 1 and 2 are shown at left, representing slightly different spectra from the colloidal Au sample. Both spectra were used to “unmix” the final images. Each pixel is 0.02 µm and the image dimensions are 21.42 µm×21.42 µm. **B.** Spectral image of hair buried for 6 months under Experiment 4 conditions (experiment 4 = hair + Au + bacteria in bacterial growth medium) displayed as “Lambda Coded”. The spectral information of pixel locations 1, 2, 3, and 4 are shown at left. Pixel location 1 is auto-fluorescence of the hair. Pixels 2 and 3 are different spectra of Au and 4 is background pixel. (ROI = region of interest).

**Figure 3:**
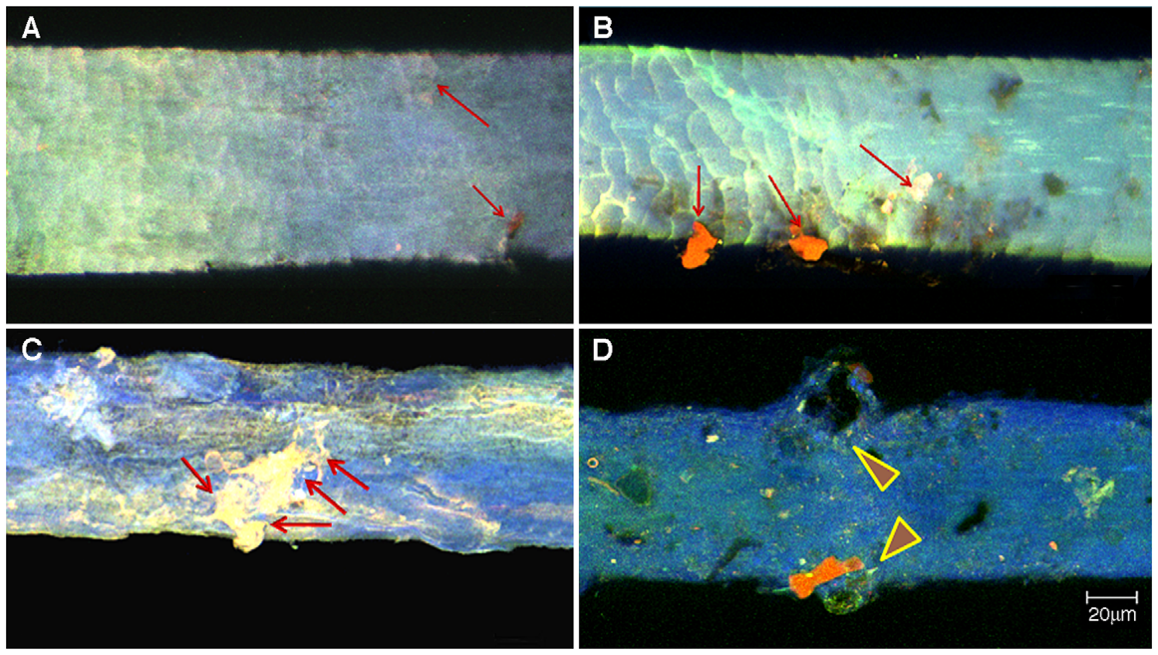
Confocal images of single hairs harvested at the end of the experiment, after six months, illustrating the effects of the experimental conditions. A. Experiment 1, hair buried in undisturbed soil cores. B. Experiment 2, hair buried in sieved (<2 mm) soil with 10 µL of 0.5 M AuCl_4_^−^ (for 385 g d.w. soil) to assess if additional Au complexes added to the soil could increase the deposition of Au on the hair. **C.** Sieved (<2 mm) soil with 10 µL of 0.5 M AuCl_4_^−^ (for 385 g d.w.) and additional *C. metallidurans* bacterial cells (1 mL of cell suspension containing 10^10^ cells mL^−1^) to assess the effect of bacterial cells and additional gold in the soil, on gold deposition in the hair. **D.** Hair samples incubated in 500 mL growth medium (1∶1 peptone meat extract broth, Oxoid), inoculated with *C. metallidurans* cells, with added 3 µL of 0.5 M AuCl_4_^−^ to assess the effect of an optimal growth medium for the bacterium and additional Au; on Au deposition on the hair. Note the increased numbers of biofilms, A–C (arrows). In D, experiment 4, hair incubated in growth medium, the biofilms have damaged the hair shaft (arrowheads). Notable is the focal accumulation bacterial biofilms on the hair shafts. This localized accumulation of bacteria and metal precluded quantification of Au content of the samples and necessitated analyzes by ICP-MS. (Scale bar applies to all panels).

**Figure 4:**
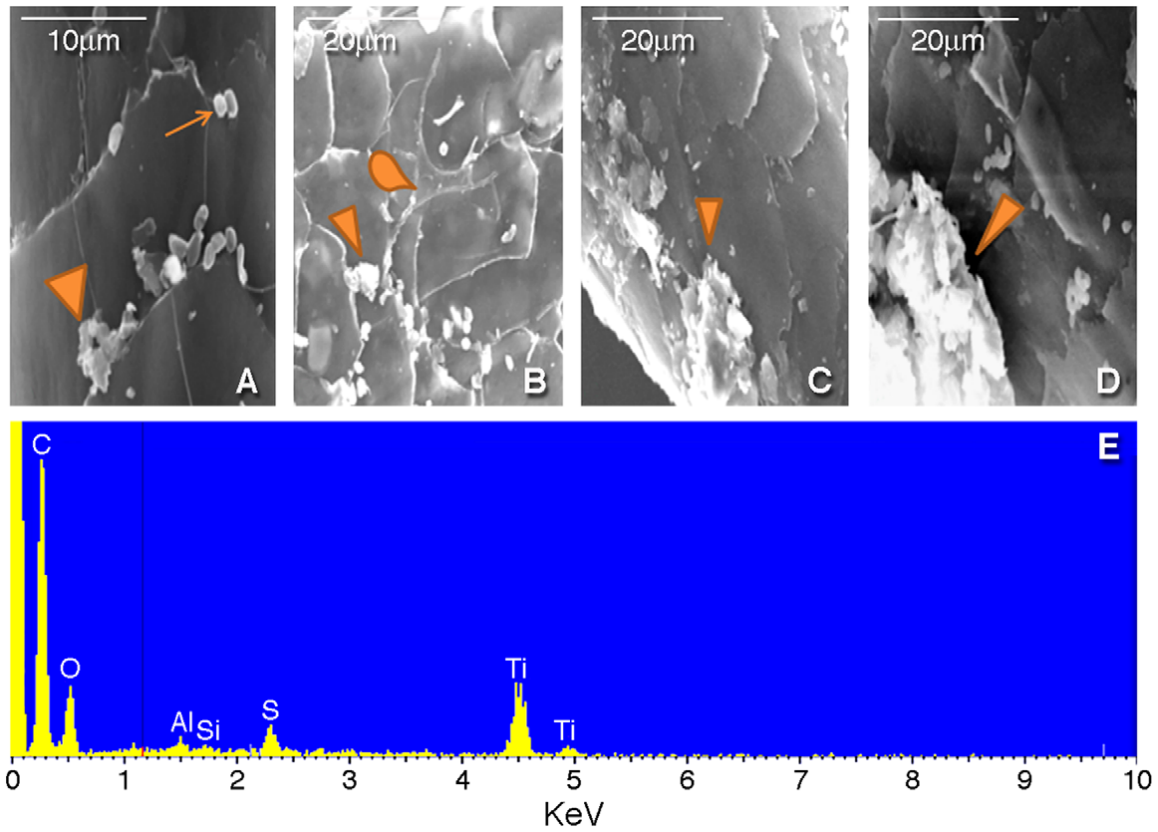
Scanning electron microscopic (SEM) picture and Energy Dispersive X-ray Spectroscopy (EDS) of a single hair from experiment 4, hair incubated in growth medium with added Au and bacterial cells, for 6 months. **A.** Single bacterium (arrow) and bacterial clumps (arrow head). The scale like structures in the background throughout all images (A–D) is normal hair cuticle, the outer covering of the hair shaft. **B.** Fungal structure (drop) and bacterial clumps with metal deposition (arrow head). **C.** larger clumps of bacteria (biofilm) (arrow head). **D.** Large accumulation of bacteria and metal (biofilm) (arrow head). **E.** EDS of the same hair showing the presence of Titanium (Ti), Sulfur (S), Silica (Si), Aluminum (Al), Oxygen (O) and Carbon (C); no Au was detected. The elemental Au content of this hair was below the detection limits of the machine. The most likely source of Ti on the hair is contamination from hair shampoo and sun-block screens, all of which contain Ti-oxide.

To verify the presence of Au on the hair, we obtained spectral images from 10 nm colloidal Au particles (Fig 2) and matched these spectra with those obtained from hair immersed in PME with added bacterial cells and Au(III)-complexes (Experiment 4), because this experiment showed the highest level of Au deposition in hair (Fig 2). This analysis, suitable for the examination of small particles on individual hairs, confirmed the deposition of Au particles on this hair. Confocal images of hair harvested after six months show the accumulation of rod-shaped bacterial-metal clumps on the hair shafts, as shown for Experiments 1 to 4 in Fig. 3. Scanning electron microscopic (SEM) image and Energy Dispersive X-ray Spectroscopy (EDS) of a single hair is shown in Fig. 4. Single bacteria and clumps of bacteria/metal-biofilms are shown localized at various levels of this hair shaft.

The S content of the hair did not change, with time, or with ordered experiment (main effect, *P* = 0.38). Notably, the ratios of Au to S increased with time (linear trend *P* = 0.02) and with ordered experiment (linear trend, *P* = 0.005). This is consistent with bacterial activity being responsible for the change in Au content in the hair as found in these experiments.

## Discussion

We report the results of experiments conducted over a period of six months in two continents using human hair buried in different soils and varying, manipulated, soil microflorae. Our analyses provide the first evidence of increasing Au deposition on hair, with time, with increasing bacterial cell-numbers and increasing Au content in the soil and with improved growth conditions for bacteria (Experiments 1–4). Hence, we consider these results relevant to archeological, forensic and toxicological investigations, in which hair is used as a proxy for metabolic processes during life.

Experiment 1 (unamended, auriferous soil) showed an increasing trend of Au deposition in hair over 6 months, yet statistical significance could not be established. This indicates that in this experiment, Au accumulation in hair occurred in natural auriferous soils, but that insufficient time, low rates of Au mobilization, lack of sufficient numbers Au precipitating bacterial cells, metabolic impairment of bacterial cells, a combination of these factors or additional unknown influences may have limited the amount of Au deposited in hair. In Experiments 3 (auriferous soils amended with additional Au(III)-complexes and bacterial cells) the Au content increased rapidly during the first three months after which it remained stable. In contrast, in Experiment 2 (auriferous soils amended with Au(III)-complexes, only) a steady increase in Au deposition was observed over the duration of the experiment. The concentration of Au(III)-complexes added to the soils was identical in these experiments, hence the difference in uptake rate is likely the result of the reductive precipitation of Au(III)-complexes in a biofilm formed by *C. metallidurans*. An earlier study has shown that the presence of aqueous Au-complexes triggers rapid, active detoxification via efflux and reductive precipitation in *C. metallidurans*, which is mediated by two Au-specific operons [15]. *C. metallidurans* rapidly accumulates Au(III)-complexes from solution [15]. Bulk and microbeam synchrotron X-ray analyses revealed that cellular Au accumulation is coupled to the formation of Au(I)-S complexes [15]. This process promotes Au toxicity and *C. metallidurans* reacts by inducing oxidative stress and metal resistances gene clusters (including an Au-specific operon) to promote cellular defence. As a result, Au detoxification is mediated by a combination of efflux, reduction, and possibly methylation of Au-complexes, leading to the formation of Au(I)-C-compounds and colloidal Au [15]. Hence, *C. metallidurans* appears to have an evolutionary advantage by being able to detoxify high concentrations of mobile Au early during exposure and thus being able to survive in highly auriferous micro-environments such as Au grain surfaces [16]. Similar mechanisms allow other bacteria to survive and flourish in toxic sludges where they have been used for clean-up of heavily contaminated soils [16], [17].

Results of Experiments 2 and 3 are further supported by the results of Experiment 4 (hair incubated in PME medium amended with *C. metallidurans* and Au(III)-complexes). Here the highest total amount of Au uptake was observed after six months, and similarly to Experiment 3 the highest rate of Au deposition was observed between month one and three, with slower rates of deposition occurring between months three and six. Despite the amendment of soils with *C. metallidurans* the total concentration of Au in hair in Experiments 2 and 3 is similar after 6 months, suggesting that (i) a steady state of Au deposition has been reached and (ii) that the natural microbial community may have adapted to higher Au concentration. These results have far-reaching implications for environmental systems in particular burial sites at crime scene, where the victim has been buried for short periods of time, *i.e.*, days to months. In this situation, concentrations and activity of mobile heavy metals and composition of the microbial community may be highly variable depending on conditions, such as season, climate, rainfall, and nutrient input. As a result, concentration of metals in hair may be highly variable even within the same site. In contrast, at archeological sites or sites where the victim has been buried for longer periods an increased metal content compared to pre-burial hair is expected, but intra-site variability may be reduced.

Sulfur is a natural component of hair, which contributes approximately 5 wt. % of mass of human hair (Fig. 1). The outer tough membrane of human hair, the cuticle, consists of several layers of flat cells containing tough, S-rich KAP5 and KAP10 proteins that protect the hair interior from the environment [18]. These proteins are also cross-linked by disulphide bonds, which increase the resistance of hair to microbial attack and decomposition [18]. In our experiments the S content, which is a reflection of living hair and its metabolism, should have remained unchanged for the short duration of this experiment once the hair had been cut, removed from its roots and buried in soil. Our results confirmed this assumption. Hence, we used the S-content as a measure of hair metabolism. We then analyzed the effects of time and of our experiments on the Au-to-S ratios and found that these ratios increased with time of burial (linear trend *P* = 0.02) and with ordered experiment (Experiments 1–4; linear trend, *P* = 0.005). This further supports our contention that the activity of *C. metallidurans* led to the increasing Au content in the hair.

Based on our experiments we conclude that soil biota can significantly change the results of toxicological analysis and impact archeological, forensic and toxicological evidence. This conclusion is further supported by a study of hair from two mummies from different locations in South America, which indicated that bacteria that enter the hair *post-mortem* can account for high levels of toxic elements on subsequent analysis. [8]. In this study, confocal microscopy of these mummified hairs did reveal localized biofilms in association with metal clumps, and the ICP-MS analysis showed high levels of elements consistent with the different heavy metal contents and biota of the two burial sites [8]. Thus, in archeological, forensic and toxicological examinations of hair, soil biota and toxic metal content are important determinants of results with wide ranging implications for interpretation of hair analyses and should be taken into account in any investigations of buried hair. Additional steps should be taken to ensure “best practice” [such as Scientific Working Group on Material Analysis (SWGMAT)] guidelines. Hence, hair should, routinely, be examined using SEM-EDS as well as bacterial 16S rRNA PCR after washing, to assess if bacterial biofilms (focal accumulations of products of bacterial metabolism that act as protective shields for the organism and accumulate toxins injurious to the cell) are present and their capacity to accumulate heavy metals. As illustrated in our confocal and SEM figures (Figs. 3, 4) microbial deposition of metals on hair are localized to accumulations in biofilms. By contrast, metabolic activity, responsible for metal accumulation during life, would be expected to be more uniformly distributed along hair shafts.

It is becoming abundantly clear that physiology, health and disease depend heavily on skin surface and gut microflorae. The genetic interactions between human genes and that of the bacterial communities in the surroundings determine our metabolic processes.[19] In turn, the analysis of chemical finger prints in the hair left by metabolic processes, after burial, such as we reported here, is complicated by biota surrounding the organisms or specific tissues such as hair. These important insights apply also to archeological, forensic and toxicological investigations which can be affected, as we have shown, by biota in the soil.

## Materials and Methods

### Ethics Statement

The NMHEMC Institutional Review Board approved this study and the subject (OA) gave written informed consent. The study was conducted according to the principles expressed in the Declaration of Helsinki.

### Hair Burial Experiments

Hair for this study was obtained from one individual (OA) at one time; this hair was used for all experiments. A smaller amount was stored for later analysis without burial in soil; the baseline sample (Fig. 1; Table S1; Fig. S1).

Soil cores used for experiment 1 were obtained from the Tomakin Park Gold Mine. Sieved (<2 mm) horizon soil for experiments 2 and 3 were collected from auriferous soils overlying the Tomakin Park Gold Mine. Separate hair samples were buried in the soils and maintained in Australia throughout the experiments. The soils were incubated at 80% WHC (water holding capacity) for one, three and six months at 25°C using a day/night regime of 16 h light and 8 h dark (Text S1). Five different experiments were performed (Fig. S1).

***Experiment 1*** : Hair was buried in undisturbed soil cores containing approximately 100 µg of Au kg^−1^ of soil. ***Experiment 2*** : Hair was buried in sieved (<2 mm) soil with 10 µL of 0.5 M AuCl_4_^−^ (for 385 g d.w. dry weight soil) to assess if additional Au complexes added to the soil would increase the deposition of Au on the hair. ***Experiment 3*** : Sieved (<2 mm) soil was used for this experiment with 10 µL of 0.5 M AuCl_4_^−^ (for 385 g d.w.) and additional *C. metallidurans* cells (1 mL of cell suspension containing 10^10^ cells mL^−1^) to assess the effect of cells and additional Au in the soil, on Au deposition in the hair. ***Experiment 4*** : Hair samples were incubated in 500 mL growth medium (1∶1 peptone meat extract (PME) broth, Oxoid), inoculated with *C. metallidurans* cells, with added 3 µL of 0.5 M AuCl_4_^−^ to assess the effect of an optimal growth medium for the bacterium and additional Au, on Au deposition on the hair. In a control experiment ***Experiment 5*** : part of the hair was analysed without burial in non-auriferous backyard soil in Albuquerque NM USA, and part was buried for one and three months (Table S1).

A fresh replicate was established for each sampling time and for each experiment. In each of the 4 experiments in Australia the hair was extracted after 1, 3 and 6 month of incubation. The samples were manually cleaned from adhering soil and sent for analyses to the United States of America (Text S1; Fig. S1).

### Field Site Description

The study area lies in the Molong - South Coast Anticlinorial Zone, which is a structural subdivision of the Lachlan Fold Belt. Gold-silver, arsenopyrite, and pyrite vein deposits form the largest and richest group of ore deposits in the area. The Tomakin Park Gold Mine is located 2 km west of the coastal village of Tomakin at S 35°48′51.9″ and E 150°10′26.4″ in New South Wales, Australia. The mine was developed in 1933 and worked until 1939 (Text S1).

### Hair Analysis

The hair samples for inductively coupled plasma-mass spectrometry (ICP/MS) were washed with Triton X-100 and rinsed with deionized water, followed by a wash with 0.05 N HNO3 and finally several more rinses in deionized water. Samples were allowed to dry at room temperature and transferred to storage before analysis.

Hair samples were then weighed and digested in 5 mL nitric acid, at 90°C. After digestion was completed, digests were brought up to 10 mL final volume and transferred into ICP plastic tubes. ICP-MS was used to analyze the samples following US EPA method 200.8. Perkin Elmer DRC II ICP/MS in which two gasses (oxygen and anhydrous ammonia) were used in the Dynamic Reaction Cell (DRC) to separate interferences. Results were then calculated using the starting weight of the hair samples and the final volume after digestion (10 mL). Results were expressed as µg kg^−1^-equivalent to parts per billion (ppb).

### Confocal Microscopy (CF)

Samples were prepared for CF by cutting hair segments and mounting them in Prolong Au mounting medium (Invitrogen) on a microscope slide under a #1.5 coverslip. Images show only intrinsic fluorescence of the samples; no exogenous fluorescent labels were added. Images were acquired on a laser scanning Zeiss LSM510 META confocal system with a 63×/1.4 NA oil immersion objective for the spectral images, and a 40×/1.3 NA oil immersion objective for the non-spectral images.

Some confocal images were acquired with the LSM510 software in “lambda” (spectral) mode. Intrinsic fluorescence was excited with a 543 µm helium neon laser. Fluorescence emission was collected across a 171 nm spectrum by a 32-channel array detector with 10.7 nm resolution (561 nm–732 µm). A control spectrum for Au was collected by imaging 10 µm colloidal Au. Two slightly shifted peaks were found which were both used as Au control spectra to “unmix” sample images. Intrinsic fluorescence of the hair varied depending on location in the sample. Five different auto-fluorescence spectra were identified. These, along with the two Au control spectra were used to “unmix” spectral images of the Au on the hair samples. Additional information on spectral imaging and linear “unmixing” can be found in reference [20].

Other confocal images were acquired using “Channel Mode” (non-spectral) of the LSM510 software. Sample fluorescence was excited and collected sequentially in three PMT channels: 405 µm diode laser excitation/420–480 µm emission; 488 µm Ar laser excitation/505–530 µm emission; 543 µm helium neon laser excitation/561–657 µm emissions. The resulting channel images are displayed as a merged image.

### Scanning Electron Microscopy (SEM)

In SEM an electron beam is scanned across the specimen. The instrument detects specific signals which produce an image of the specimen and/or a record of the sample's elemental composition; Energy Dispersive X-ray Spectroscopy (EDS). Single hairs were submitted to the SEM.

### Statistical Methods

Descriptive data obtained by ICP-MS were reported as mean ± SD or frequency (%). The main analysis is a 2-way ANOVA of each outcome (the corrected concentration of Au and of S, for the weight of each hair sample and their ratio; gold/sulfur ratios) with months (1, 3 and 6) and ordered experiments (Experiment 1, 2, 3 and 4) as the two factors. With one observation per cell of the design layout, month and experiment were treated as linear factors in the ANOVA; and with no significant interaction, the additive model was used. These assumptions were checked graphically. Since small quantities of Au were present in the hair before burial and these amounts did not change after burial in non-auriferous soil in the USA for up to three months, we subtracted the mean (52.8 µg kg^−1^of hair) from all values obtained after burial of the hair in Australian auriferous soil. All statistical analyses were performed in SAS 9.2; *P*-values≤0.05 were considered statistically significant.

## Supporting Information

Table S1 Gold (Au) and sulfur (S) quantities obtained by ICP/MS and description of experiments.
(0.04 MB DOC)

Text S1 Supplementary Materials and Methods.
(2.48 MB DOC)

Figure S1 Schematic representation of experiments and sampling times; Australia and USA.
(0.43 MB TIF)
