# Supplementary material for: Bacterial Deposition of Gold on Hair: Archeological, Forensic and Toxicological Implications
Source: PLoS One. 2010 Feb 19;5(2):e9335. doi: 10.1371/journal.pone.0009335 (PMC2824836; doi:10.1371/journal.pone.0009335)
Supplement: Table S1 — Gold (Au) and sulfur (S) quantities obtained by ICP/MS and description of experiments. (0.04 MB DOC) [file pone.0009335.s001.doc]

| **Experiment [Number]**  **New South Wales, Australia** | **Time [Month]** | **Au [µg Kg^-1^]** | **S [µg Kg^-1^]** |
| --- | --- | --- | --- |
| **1** | **1** | **114** | **54450** |
| **2** | **1** | **114** | **15880** |
| **3** | **1** | **237** | **7507** |
| **4** | **1** | **492** | **13464** |
|  |  |  |  |
| **1** | **3** | **189** | **14424** |
| **2** | **3** | **439** | **14502** |
| **3** | **3** | **699** | **16413** |
| **4** | **3** | **1454** | **13077** |
|  |  |  |  |
| **1** | **6** | **179** | **10779.7** |
| **2** | **6** | **661** | **8650.6** |
| **3** | **6** | **689** | **9231.9** |
| **4** | **6** | **1619** | **8080.2** |
|  |  |  |  |
| **ABQ Base.** | **0** | **64.9** | **12740** |
| **5** | **1** | **39.9** | **11399** |
| **5** | **3** | **53.6** | **7679** |

**Table : Showing gold (Au) and sulfur (S) quantities obtained by ICP/MS.**

Experiment number and timing are indicated. ABQ= Albuquerque NM USA. All experiments involved burial of hair in soil, except experiment 4 =hair samples incubated in growth medium and ABQ Base in which the hair was analyzed without prior burial in soil.

***Experiment 1***: Hair was buried in undisturbed soil cores containing approximately 100 µg of Au kg^-1^ of soil. ***Experiment 2***: Hair was buried in sieved (< 2 mm) soil with 10 µL of 0.5 M AuCl_4_^-^ (for 385 g d.w. dry weight soil) to assess if additional Au complexes added to the soil would increase the deposition of Au on the hair. ***Experiment 3***: Sieved (< 2 mm) soil was used for this experiment with 10 µL of 0.5 M AuCl_4_^-^ (for 385 g d.w.) and additional *C. metallidurans* cells (1 mL of cell suspension containing 10^10^ cells mL^-1^) to assess the effect of cells and additional Au in the soil, on Au deposition in the hair. ***Experiment 4***: Hair samples were incubated in 500 mL growth medium (1:1 peptone meat extract broth, Oxoid), inoculated with *C. metallidurans* cells, with added 3 µL of 0.5 M AuCl_4_^-^ to assess the effect of an optimal growth medium for the bacterium and additional Au, on Au deposition on the hair. In control experiment ***Experiment 5*** Part of the hair was analysed without burial in non-auriferous backyard soil in Albuquerque NM USA, (ABQ Base) and part was buried for one and three months.
