# Supplementary material for: Bacterial Deposition of Gold on Hair: Archeological, Forensic and Toxicological Implications
Source: PLoS One. 2010 Feb 19;5(2):e9335. doi: 10.1371/journal.pone.0009335 (PMC2824836; doi:10.1371/journal.pone.0009335)
Supplement: Text S1 — Supplementary Materials and Methods. (2.48 MB DOC) [file pone.0009335.s002.doc]

**Text S1**

**Supplementary Materials and Methods**

***Field site description***

*Geological setting*

The geological, mineralogical and metallogenic description of the study area is summarised after McIlveen (1975) [1]. The study area lies in the Molong - South Coast Anticlinorial Zone, which is a structural subdivision of the Lachlan Fold Belt. Gold-silver, arsenopyrite, and pyrite vein deposits form the largest and richest group of ore deposits in the area (*i.e.*, on the Ulladulla 1:250000 map sheet; McIlveen, 1973 [2]. The vein deposits occur either within acid intrusive bodies of the Moruya complex or in the Middle-to-Late Ordovician metasediments surrounding the intrusions. They are regarded as hydrothermal quartz vein deposits derived from late stage acid differentiates of the Moruya Batholith. The deposits are generally formed by simple quartz veins from several cm to 1 m in width, and occur in groups or as individual veins. The Tomakin Park Gold Mine is located 2 km west of the coastal village of Tomakin (Fig. 1) at S 35°48’51.9’’ and E 150°10’26.4’’, respectively. The Ulladulla metallogenic 1:250000 map-sheet shows the mine as deposit 54 [2] It occurs in portion 64, Bateman Parish, St. Vincent Shire [1] The mine was developed in 1933 and worked until 1939. The main shaft was sunk 12 m and approximately 27 m of driving was completed (Fig. 2 A). Metamorphosed interbedded greywacke, shale and siltstone of Middle to Late Ordovician age crop out in the area. The lithologies are a typical flysch wedge sequence. Scheibner (1976) [3] interpreted the sediments as having been deposited in an arc-trench gap, *i.e.*, the Monaro Slope and Basin. The rocks strike north-easterly and dip vertically to steeply southeast. Cleavage is approximately parallel to bedding. The worked Au quartz vein crops out for about 70 m northward from immediately south of the workings. It strikes variably around 035° and dips about 75° W. The vein is on the hanging wall of the open stope [4] and varies in thickness from a few centimetres at the southern end of the open stope to 0.3 m at the northern end. The quartz vein is vuggy with numerous crystal-lined voids. Pyrite crystal aggregates are present in the unweathered quartz. In the primary ore, the Au occurs within the arsenopyrite and pyrite in solid solution or as small inclusions. However, the quartz vein material is mostly weathered and contains limonite, rather than fresh pyrite. To the east of the major Au bearing quartz vein, several smaller Au bearing quartz veins have been located. These strike in the same general direction but appear to be much poorer in Au [4].

**
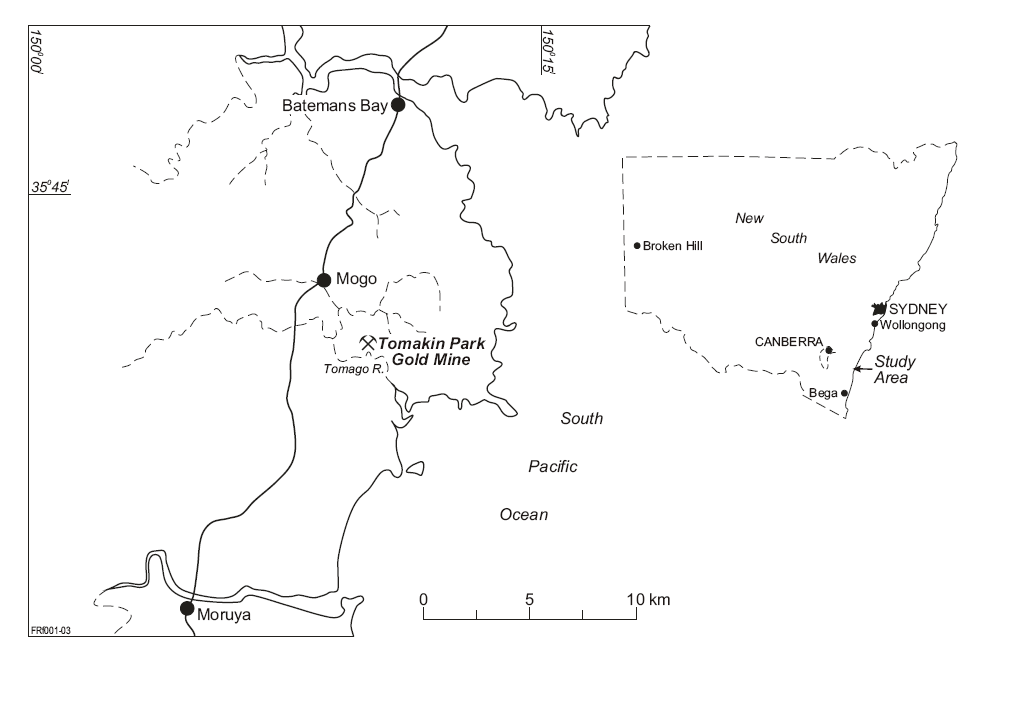
**

**Figure 1** Location map of the Tomakin Park Gold Mine in south eastern New South Wales, Australia.

**Climate, geomorphology, soil and vegetation**

The climate in the region is temperate due to its proximity to the Pacific Ocean. Maritime influence leads to a uniformly distributed rainfall of about 1000 mm per annum. Mean maximum summer temperatures and mean minimum winter temperatures are approximately 20°C and 6°C, respectively, and freezing seldom occurs in the study area. The study area lies approximately 10 to 60 m above sea level. The landscape of the study area is characterised by narrow ridgelines with gentle to very steep side slopes. The topography of the study area is depicted in Figure 2.1C. The Tomaga River is located 50 m south of the entrance to the mine. An alluvial plain is located on the opposite bank of the river. The weathered cover is up to several metres in depth. Exposed profiles in the mineshafts and drifts are slightly, to moderately weathered (Fig 2b). The study area is covered by an open *Eucalypt* forest (*Eucalyptus maculata* (Spotted Gum), *E. paniculata* (Grey Ironbark), *E. pilularis* (Blackbutt), *Callitris columellaris* (Cypress-pine), *E. gummifera* (Red bloodwood) and *E. sieberi* (Silvertop ash), with patches of rainforest in the gullies. The cycad *Macrozamia communis* is a common understorey shrub. Field analyses of soils, after McDonald *et al.* (1998) [5], were conducted on top of the ridge, at the bottom of the ridge close to the mine entrance, and on the gentle slope where the quartz vein crops out (Fig. 2b). The soils are colluvial, and the soil type is a brown soil or cambisol. In some areas a slight podsolisation is visible. A typical soil profile (thickness) consisted of a 1-3 cm O-horizon, a 3-12 cm Ah-horizon, a 15-20 cm B_1_-horizon and a 50-100 cm B_2_-horizon followed by a moderately weathered saprock C-horizon at greater depth. The soil textures are predominantly sandy or silty clay loams with gravel contents of up to 30 vol.%. A few (< 2 vol.%) mottles were detected in the B-horizons and were orange to pale brown of colour.

**A**

**B**

**
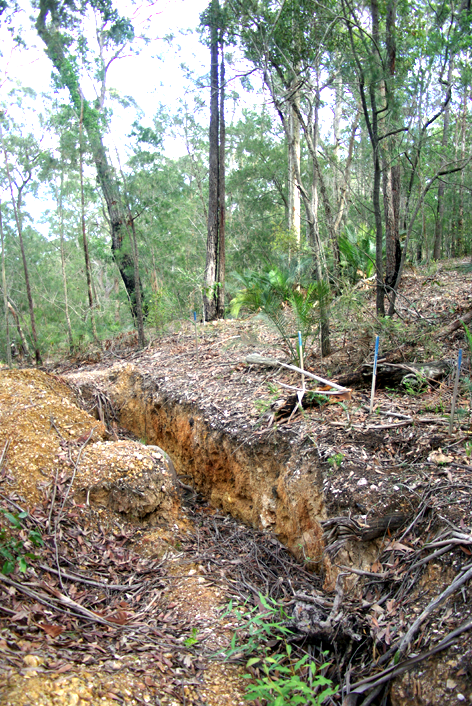
**


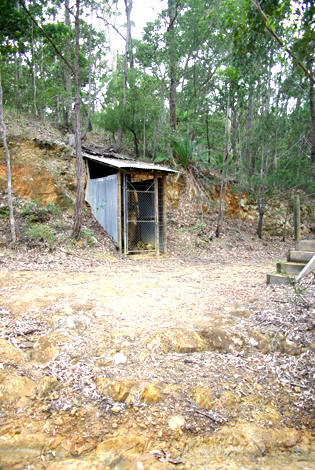


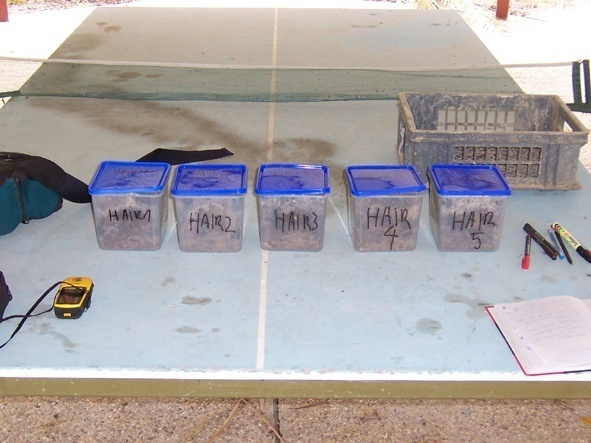


**C**

**Figure 2**

Collection of soil samples for hair burial experiments at the Tomakin Park Gold Mine. (A) Mine entrance; (B) overlying trench; (C) Collected soil cores for Experiment 1.

**Experimental approach**

***Hair burial experiments***

Cores for Experiment 1 (Fig. 2 c,) and sieved (< 2mm) Ah horizon soil for experiments 2 and 3 (Fig. 3b) were collected from auriferous soils overlying the Tomakin Park Gold Mine on the 26.3.2007. Hair samples were supplied by O. Appenzeller and buried in the soils. Soils were incubated at 80% WHC (water holding capacity) from 1, 3 and 6 months at 25ºC using a day/night regime of 16 h light and 8 h dark. Four different experiments were performed: Experiment 1: Soil was buried in undisturbed soil cores. Experiment 2: Sieved (< 2 mm) was used for the experiments, which was amended with 10 µL of 0.5 M AuCl_4_^-^ (for 385 g d.w. dry weight soil) to assess if additional Au complexes buried in soil would increase the deposition of Au in the hair. (Fig. 3b). Experiment 3: Sieved (< 2 mm) was used for the experiments, which was amended with 10 µL of 0.5 M AuCl_4_^-^ (for 385 g d.w. dry weight soil) and additional *Cupriavidus metallidurans* cells (1 mL of cell suspension containing 1010 cells mL^-1^) to assess the effect of cells and additional Au on Au deposition in the hair buried in soil (Fig. 3b). Experiment 4: Hair samples were incubated in 500 mL growth medium (1:1 peptone meat extract broth, Oxoid) inoculated with *C. metallidurans* and amended with 3 µL of 0.5 M AuCl_4_^-^ to assess the effect of cells and additional Au on Au deposition in the hair (Fig. 3 c,d).A fresh replicate was established for each sampling time for each experiment, and hair was extracted after 1, 3 and 6 month of incubation. Hair was manually cleaned from adhering soil and send for analyses to the USA.

***Geochemical soil analyses using XRD and ICP-MS***

Minerals (Table 1) were identified and their abundances determined at Geoscience Australia using XRD on powdered samples and the SIROQUANT software. following the procedures outlined in Taylor (1991) [6]. Total carbon, C_tot_, and total nitrogen, N_tot_, contents of Ah- and B-horizon samples were measured using a Leco CNS 2000 element analyser. The pH values of soil (in 1:5 soil water) were measured with an Activon pH electrode and meter. Size fraction analysis of the < 2mm Ah- and B-horizon samples was conducted using the pipette method (USDA Method 3A1, 1996). For analyses of major, minor and trace elements 0.5 g of soil were extracted in triplicate following the US EPA method 3051a. A 3:1 HCl:HNO_3_ acid digestion making up to a final volume of 50 ml with MilliQ H_2_O. The digests were carried out using a 12-rotor Milestone Ethos E microwave. Element concentrations in the digests where then established using Inductively Couple Plasma Mass Spectometry (ICP-MS). The ICP-MS used was an Agilent 7500ce (Agilent Technologies, Tokyo, Japan) connected to a CETAC ASX-500 autosampler. Samples were run using helium as a collision gas to remove oxide or doubly charged mass interferences on the 197 mass to be detected. The approximate quantification limit for Au was 0.4 ppb.


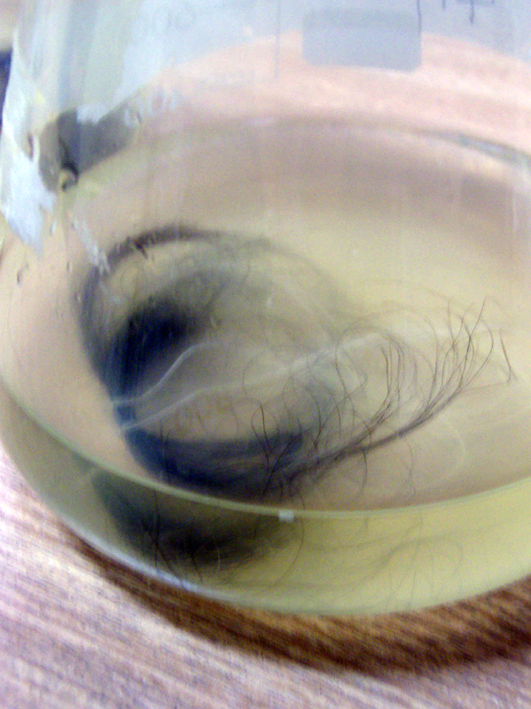


**A**

**C**


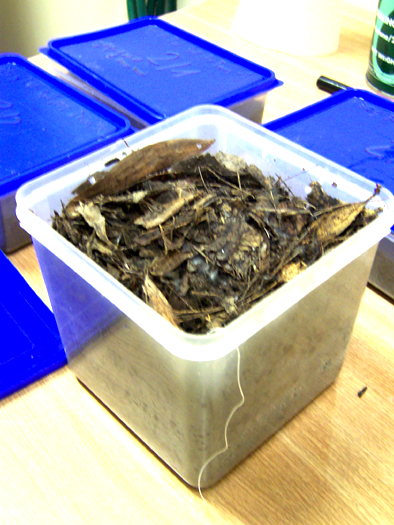


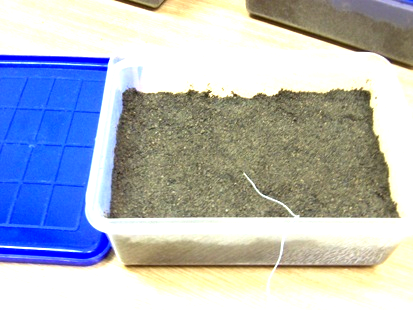


**B**

**Figure 3**

D

Experimental setup in the laboratory. (A) Experiment 1 with buried hair (B) buried hair in Experiments 2 and 3. (C) Experiment 4.

C

**Geochemical analyses of the soil used for hair burial experiments**

Mineralogy and other soil properties are shown in Table 1. In Table 2 the concentrations of major, minor and trace elements in microwave digests of the soils are shown.

**Table 1**

Mineralogy and properties of the auriferous soil and used in the hair burial experiments 1, 2 and 3 (from Reith et al., 2005) [7]

|  | **Ah-horizon** | **B-horizon** |
| --- | --- | --- |
|  |  |  |
| **Mineralogy**  [%] |  |  |
|  |  |  |
| Quartz | 80.1 | 47.3 |
| Albite | 1.3 | 1.2 |
| Muscovite | 12.2 | 42.5 |
| Kaolin | 1.0 | 5.1 |
| Illite | 0.2 | 0.2 |
| Goethite | 0.4 | 1.3 |
| Microcline | 2.1 | n.d. |
| Halloysite | 0.2 | n.d. |
| Pyrophillite | n.d.^a^ | n.d. |
| Biotite | n.d. | 1.4 |
| Rutile | n.d. | 1.0 |
|  |  |  |
| **Size Fraction Analysis** [%] |  |  |
| Coarse Sand | 23 | 24 |
| Fine Sand | 30 | 20 |
| Silt | 25 | 25 |
| Clay | 11 | 32 |
|  |  |  |
| Total C [wt. %] | 9.6 | 0.7 |
| Total N [wt. %] | 0.33 | 0.09 |
| pH _(1:5 extract in water)_ | 5.5 | 6.0 |
|  |  |  |

**^a^ n.d., not detected**

**Table 2**

Content of major, minor and trace elements in the auriferous soil from the Tomakin Park Gold Mine, NSW, Australia, and non-auriferous soil from Albuquerque, NM, USA soil used for hair burial experiments. Shown are averaged and standard deviation (±) of 3 replicate analyses (Australia) and 1 replicate (Albuquerque, NM, USA).

| Element Concentration | **Tomakin, Australia**  **Ah-horizon** | **Tomakin, Australia**  **B-horizon** | Albuquerque **Soil, USA** |
| --- | --- | --- | --- |
|  |  |  |  |
| **Majors [g Kg^-1^]** |  |  |  |
|  |  |  |  |
| Al | 25.3 ± 1.0 | 33.1 ± 3.9 | 7.1 |
| Ca | 1.6 ± 0.7 | 0.5 ± 0.2 | 5.8 |
| Fe | 11.8 ± 1.7 | 13.5 ± 1.8 | 10.7 |
| K | 9.8 ± 0.2 | 12.1 ± 1.3 | 2.6 |
| Mg | 1.8 ± 0.2 | 1.9 ± 0.2 | 3.2 |
|  |  |  |  |
| **Minors [mg Kg^-1^]** |  |  |  |
| As | 126.8 ± 30.3 | 170.0 ± 60.1 | 1.7 |
| B | 13.3 ± 5.8 | 20.0 ± 0.5 | n.a. |
| Co | 10.0 ± 1.4 | 10.0 ± 0.5 | n.a. |
| Cr | 21.7 ± 2.9 | 28.3 ± 2.9 | 2.5 |
| Mn | 286.6 ± 48.5 | 138.4 ± 41.6 | 294.0 |
| Na | 330.0 ± 88.9 | 353.3 ± 28.9 | 230.0 |
| P | 170.0 ± 30.0 | 123.3 ± 5.8 | n.a. |
| Pb | 42.7 ± 16.1 | 41.7 ± 25.7 | 5.1 |
| S | 246.7 ± 126.6 | 123.4 ± 15.3 |  |
| Zn | 28.3 ± 5.8 | 25.0 ± 5.0 | 45.7 |
| Sc | 5.7 ± 0.7 | 6.9 ± 0.6 | n.a. |
| V | 37.1 ± 3.7 | 45.7 ± 3.4 | n.d. |
| Ni | 8.0 ± 1.1 | 7.6 ± 0.8 | 1.6 |
| Cu | 7.5 ± 0.5 | 7.3 ± 2.1 | 4.1 |
| Ga | 45.9 ± 2.5 | 49.1 ± 2.5 | n.a. |
| Sr | 37.9 ± 9.0 | 21.8 ± 1.7 | n.a. |
| Y | 7.0 ± 0.4 | 8.3 ± 0.4 | n.a. |
| Nb | 4.0 ± 0.5 | 3.8 ± 0.7 | n.a. |
| La | 47.3 ± 5.7 | 54.4 ± 5.8 | n.a. |
| Ce | 92.3 ± 11.6 | 110.6 ± 11.3 | n.a. |
| Pr | 10.2 ± 1.2 | 12.0 ± 1.1 | n.a. |
| Nd | 36.5 ± 4.4 | 42.4 ± 3.9 | n.a. |
| Sm | 6.4 ± 0.8 | 7.4 ± 0.7 | n.a. |
| Gd | 4.2 ± 0.5 | 4.9 ± 0.4 | n.a. |
| Th | 9.3 ± 1.3 | 11.0 ± 1.1 | n.a. |
|  |  |  |  |
| **Traces [µg Kg^-1^]** |  |  |  |
| Au | 130.0 ± 16.1 | 80.5 ± 2.1 | n.d. |
| Se | 514.0 ± 28.9 | 494.5 ± 64.6 | n.a. |
| Mo | 165.5 ± 12.0 | 176.1 ± 15.5 | n.a. |
| Eu | 1012.8 ± 119.5 | 1182.7 ± 116.8 | n.a. |
| Tb | 434.4 ± 42.7 | 511.6 ± 28.5 | n.a. |
| Dy | 1790.0 ± 138.2 | 2103.1 ± 89.5 | n.a. |
| Ho | 273.3 ± 17.4 | 327.2 ± 13.3 | n.a. |
| Er | 691.5 ± 39.7 | 834.5 ± 38.1 | n.a. |
| Yb | 515.9 ± 24.1 | 633.8 ± 41.2 | n.a. |
| Lu | 73.9 ± 3.1 | 89.0 ± 5.1 | n.a. |
| **W** | 307.8 ± 19.9 | 338.1 ± 5.1 | n.a. |
| Tl | 385.3 ± 27.4 | 482.5 ± 27.5 | n.a. |
| Bi | 290.0 ± 80.5 | 311.9 ± 72.1 | n.a. |
| Ag | n.d. | n.d. | n.a. |
| Cd | n.d. | n.d. | 225.0 |
| Tm | n.d. | 106.3 ± 5.1 | n.a. |
| Os |  | n.d. | n.a. |

^a^ n.d., not detected

^a^ n.a., not analyzed

**References**

1. McIlveen, G.R., (1975) Part 1. Mine data sheets to accompany Ulladulla 1:250 000 Metallogenic sheet. Part 2 A metallogenic study of the Ulladulla 1:250 000 sheet. Geological Survey of New South Wales, Sydney.
2. McIlveen, G.R., (1973) Ulladulla 1:250000 Metallogenic sheet. Geological Survey of New South Wales, Sydney.

3. Scheibner, E., (1976) Explanatory Notes on the tectonic Map of New South Wales.

Geological Survey of New South Wales, Sydney.

4. Bowman, H.N., (1979) A brief inspection of the Tomakin Park Gold Mine. Geol Surv. Development, Sydney, Australia. Gs 1979/256.

5. McDonald, R.C., Isbell, R.F., Speight, J.G., Walker, J., Hopkins, M.S., (1998) Australian Soil and Land Survey Field Handbook, Second edition. CSIRO Publishing, Australia.

6. Taylor, J.C., (1991) Computer Programs for Standardless Quantitative Analysis of Minerals Using the Full Powder Diffraction Profile. Powder Diffr. 6: 2–9.

7. Reith F, McPhail DC, Christy AG. (2005) Bacillus cereus, gold and associated elements in soil and other regolith samples from Tomakin Park Gold Mine in southeastern New South Wales, Australia. J. Geochem. Explor. 85: 81–98
