# Supplementary figures and images for: Bacterial Deposition of Gold on Hair: Archeological, Forensic and Toxicological Implications
Source: PLoS One. 2010 Feb 19;5(2):e9335. doi: 10.1371/journal.pone.0009335 (PMC2824836; doi:10.1371/journal.pone.0009335)

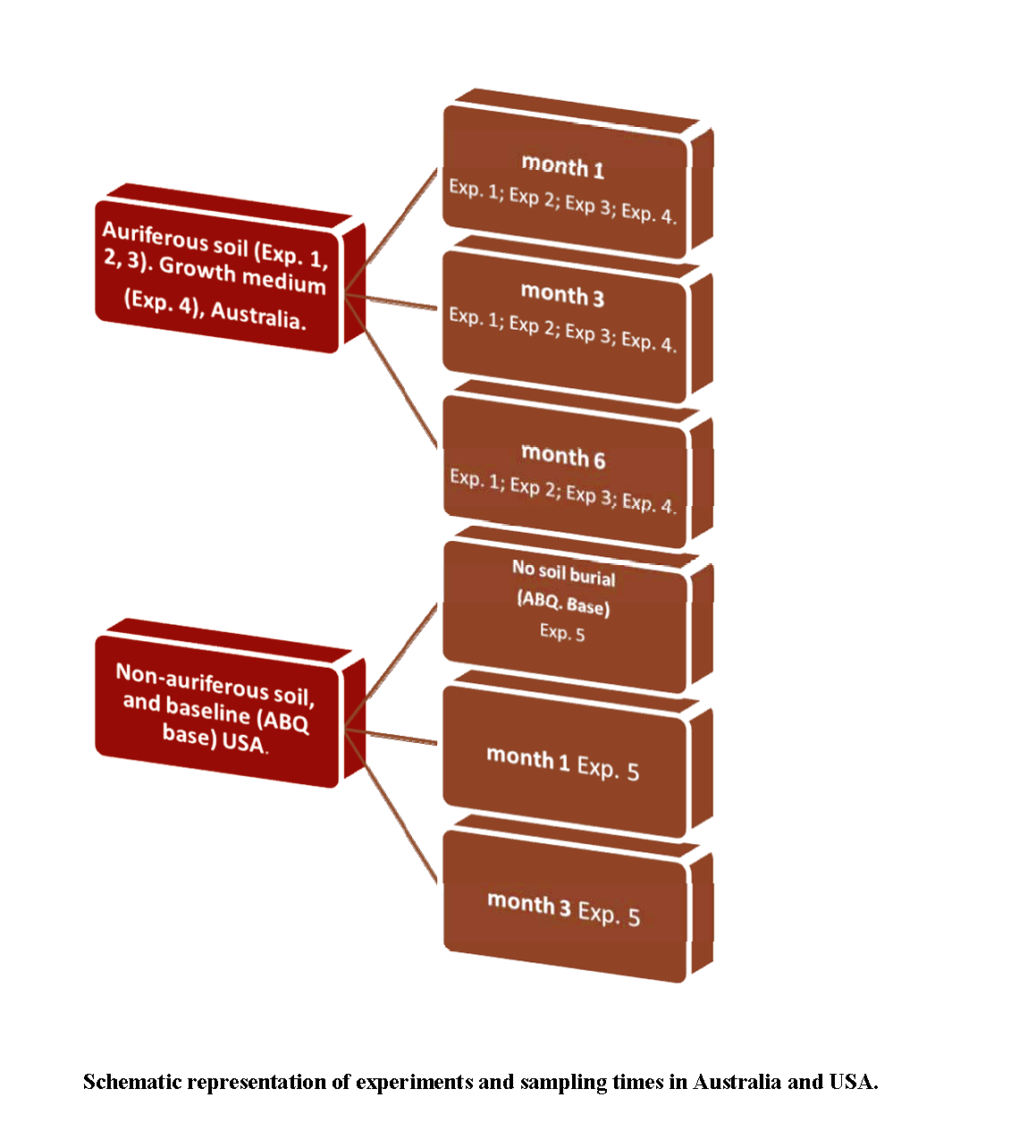

Supplement: Figure S1 — Schematic representation of experiments and sampling times; Australia and USA. (0.43 MB TIF) [file pone.0009335.s003.tif]
